# Supplementary material for: The genome sequence of the Speedwell Longhorn, Cauchas fibulella ([Denis & Schiffermüller], 1775) (Lepidoptera: Adelidae)
Source: Wellcome Open Res. 2025 Oct 21;10:594. [Version 1] doi: 10.12688/wellcomeopenres.25061.1 (PMC12754359; doi:10.12688/wellcomeopenres.25061.1)
Supplement: Peer review history

# Reviewer report: Reviewer response for version 1

**DOI:** 10.21956/wellcomeopenres.27627.r143608

**Published:** 2026-01-30

**Authors:** Sara E Miller

**Affiliations:** 1 University of Missouri at Saint Louis, St Louis, Missouri, USA

The authors present a new high quality nuclear, Z-chromosome, and mitochondrial genome assembly for the Speedwell Longhorn moth, *Cauchas fibulella *(Lepidoptera: Adelidae). The methods are consistent with other genome assemblies from the Darwin Tree of Life project and are well-described. The genome was assembled into two haplotypes. Both haplotype assemblies exceed Earth BioGenome Project Assembly Standards benchmarks. Data and code used to assemble this genome has been archived.

Are sufficient details of methods and materials provided to allow replication by others?

Yes

Is the rationale for creating the dataset(s) clearly described?

Yes

Are the datasets clearly presented in a useable and accessible format?

Yes

Are the protocols appropriate and is the work technically sound?

Yes

Reviewer Expertise:

Hymenoptera genetics and genomics, evolutionary ecology

I confirm that I have read this submission and believe that I have an appropriate level of expertise to confirm that it is of an acceptable scientific standard.

# Reviewer report: Reviewer response for version 1

**DOI:** 10.21956/wellcomeopenres.27627.r143606

**Published:** 2026-01-23

**Authors:** Oliver Rupp

**Affiliations:** 1 Justus-Liebig-University, Giessengiessen, Germany

This article presents the chromosome level genome assembly of the Speedwell Longhorn moth.

 The DNA sequencing and assembly followed well established protocols for eukaryotic genomes, including PacBio HiFi and Hi-C sequencing followed by a Hifiasm assembly and Hi-C scaffolding. The assembly includes the mitochondrial genome which was acquired using appropriate tools. The quality of the genome was assessed with different metrics, including k-mer statistics and BUSCO completeness analysis. The assembly is available in standard databases.

 Overall this article presents a high quality, chromosome level assembly.

Are sufficient details of methods and materials provided to allow replication by others?

Yes

Is the rationale for creating the dataset(s) clearly described?

Yes

Are the datasets clearly presented in a useable and accessible format?

Yes

Are the protocols appropriate and is the work technically sound?

Yes

Reviewer Expertise:

genome assembly, genome annotation

I confirm that I have read this submission and believe that I have an appropriate level of expertise to confirm that it is of an acceptable scientific standard.

# Reviewer report: Reviewer response for version 1

**DOI:** 10.21956/wellcomeopenres.27627.r143609

**Published:** 2026-01-19

**Authors:** Peng-Fe Ma

**Affiliations:** 1 Chinese Academy of Sciences, Kunming, China

This study generated a genome assembly for the longhorn moth ( *Cauchas fibulella*), which is widespread in the United Kingdom as well as in Europe.  The assembled genome is of high quality and contains two haplotypes. And the Z chromosome was also successfully assembled.  The related genomic resource will be helpful for understanding the evolution and species identification in the *Cauchas *genus.

Are sufficient details of methods and materials provided to allow replication by others?

Yes

Is the rationale for creating the dataset(s) clearly described?

Yes

Are the datasets clearly presented in a useable and accessible format?

Yes

Are the protocols appropriate and is the work technically sound?

Yes

Reviewer Expertise:

Genomics and evolution.

I confirm that I have read this submission and believe that I have an appropriate level of expertise to confirm that it is of an acceptable scientific standard.

# Reviewer report: Reviewer response for version 1

**DOI:** 10.21956/wellcomeopenres.27627.r143610

**Published:** 2026-01-13

**Authors:** Ian M Carr

**Affiliations:** 1 University of Leeds, Leeds, UK

General comments:

 I'm sure the work described here was completed to a high quality, but its write-up lets it down. For instance, the document's internal table referencing system is broken, and acronyms are used before they're described or described twice. A simple proofreading step would have improved the document significantly. I think the figures are taken from analysis software reports and just pasted in the document without any critical thinking about whether they would benefit from slight tweaks. 

 Specific comments (from the PDF version):

 The penultimate paragraph of the *Background* section discusses the genome assembly. This is a result and should be moved to a later section of the paper.

 The sentence beginning *“The assembly presented here produced using the Tree of Life pipeline”* is missing a verb. It should read: *“The assembly presented here was produced using the Tree of Life pipeline.”*

 The acronym **ToLID** is not explained on first use.

 Please define the acronym **COI** in the Methods section.

 The acronym **WSI** is introduced twice: once in the Sample Collection section and again in the Nucleic Acid Extraction section. Please remove the second introduction.

 Please provide the manufacturers for the **Qubit fluorometer**, **Nanodrop**, and **FemtoPulse** systems when they are first mentioned.

 RNA was quantified using the *Agilent RNA 6000 Pico Kit*. Please clarify whether this was run on a **TapeStation** or a **Bioanalyzer**.

 The manuscript states: *“Size selection and clean-up were performed using diluted AMPure PB beads (Pacific Biosciences).”: *AMPure beads are manufactured by **Beckman Coulter Life Sciences**, not Pacific Biosciences. If the beads were diluted, please specify the dilution factor, as bead concentration determines the size-selection cutoff.

 Figure 2 contains text beneath the GenomeScope profile tile that is not clearly explained. Although some values can be inferred from the main text, this information would be better removed from the figure and presented in a clearer format within the figure legend.

 In the *Assembly Statistics* section, the sentence *“These chromosome-level scaffolds, confirmed by Hi-C data, are named according to size (Figure 3; Table ¿tbl:table3?).”* contains a broken table reference. Please correct the link.

 Following from the last comment: There is an unlabelled table ({#tbl:table3}) between Figure 3 and Figure 4. It appears after Table 2 but before Table 3, which breaks the numbering sequence. The tables should be renumbered and labelled correctly.

 The sentence *“Z chromosome identified based BUSCO gene painting with ancestral Merian elements”* should read:

*“The Z chromosome was identified based on BUSCO gene painting with ancestral Merian elements.”*

 Figure 6 contains a large amount of empty space due to a contig with a significantly different read depth (>200) and GC content (~0.16). This contig never seems to be mentioned elsewhere.

Are sufficient details of methods and materials provided to allow replication by others?

Yes

Is the rationale for creating the dataset(s) clearly described?

Yes

Are the datasets clearly presented in a useable and accessible format?

Yes

Are the protocols appropriate and is the work technically sound?

Yes

Reviewer Expertise:

Bioinformatics and NGS data production and analysis

I confirm that I have read this submission and believe that I have an appropriate level of expertise to confirm that it is of an acceptable scientific standard.

# Reviewer report: Reviewer response for version 1

**DOI:** 10.21956/wellcomeopenres.27627.r140700

**Published:** 2025-12-30

**Authors:** Ariel Chipman

**Affiliations:** 1 Hebrew University of Jerusalem School of Pharmacy, Jerusalem, Jerusalem District, Israel

The authors present the genome sequence of the lepidopteran Cauchas fibulella, or speedwell longhorn. The genome was sequenced from two individuals collected in separate location sin England. One individual was used primary sequencing and the second for Hi-C and RNA sequencing. The protocols follow standard Darwin ToL procedures and were carried out as expected. The results are presented in a clear and standardized fashion, and don't include any surprises. The manuscript is standard and straightforward and I see no problems with its Indexing.

Are sufficient details of methods and materials provided to allow replication by others?

Yes

Is the rationale for creating the dataset(s) clearly described?

Yes

Are the datasets clearly presented in a useable and accessible format?

Yes

Are the protocols appropriate and is the work technically sound?

Yes

Reviewer Expertise:

Arthropod evolution

I confirm that I have read this submission and believe that I have an appropriate level of expertise to confirm that it is of an acceptable scientific standard.

# Reviewer report: Reviewer response for version 1

**DOI:** 10.21956/wellcomeopenres.27627.r137069

**Published:** 2025-11-10

**Authors:** Jhon Alex Dziechciarz Vidal

**Affiliations:** 1 Universidade Federal de SãoCarlos (UFSCar), São Carlos, Brazil

This manuscript presents a high-quality genome assembly for *Cauchas fibulella*, generated using the standard Darwin Tree of Life Consortium assembly pipeline. The work is well structured and contributes valuable genomic information to the growing dataset of Lepidoptera, a group for which many assemblies are already available, yet still essential for evolutionary comparative studies.

 I commend the authors for the quality of the genomic data and the clarity of the report. However, I have a few points that require clarification.

**Abstract**

 The abstract states that haplotype 1 was fully scaffolded into 25 chromosomal pseudomolecules, while 97.29% of haplotype 2 was scaffolded.

 Please indicate the equivalent percentage for haplotype 1, for consistency and clarity.

**Methods**

 The genome assembly was generated from one individual, whereas the Hi-C data originated from another. Using different individuals for primary sequencing and chromosomal scaffolding is not common and may introduce structural or heterozygosity-related inconsistencies.

 Could the authors comment on whether this approach may have affected the assembly, and if any steps were taken to mitigate potential bias?

**Figure 3**

 Although the Z chromosome is described in the text, it does not appear to be labelled or visible as a separate scaffold in Figure 3 (Hi-C map).

 Could the authors clarify why the Z chromosome is not displayed, or confirm whether it is included but not annotated in the figure?

Are sufficient details of methods and materials provided to allow replication by others?

Yes

Is the rationale for creating the dataset(s) clearly described?

Yes

Are the datasets clearly presented in a useable and accessible format?

Yes

Are the protocols appropriate and is the work technically sound?

Yes

Reviewer Expertise:

I have expertise in cytogenomics, repetitive DNA biology, and chromosome-level genome assembly, which qualifies me to evaluate this work.

I confirm that I have read this submission and believe that I have an appropriate level of expertise to confirm that it is of an acceptable scientific standard.
